# Toxicity Derived from Interaction between Natural Compounds and Cancer Therapeutic Drugs Metabolized by CYP3A4: Lessons Learned from Two Clinical Case Reports

**DOI:** 10.3390/ijms242115976

**Published:** 2023-11-05

**Authors:** Sabrina Orzetti, Paolo Baldo

**Affiliations:** 1 PCD Pharmacy Clinical Desk, Hospital Pharmacy Unit of the “Centro di Riferimento Oncologico (CRO) di Aviano IRCCS”, Via F. Gallini, 33081 Aviano, Italy; sabrina.orzetti@cro.it; 2 Hospital Pharmacy Unit of the “Centro di Riferimento Oncologico (CRO) di Aviano IRCCS”, Via F. Gallini, 33081 Aviano, Italy

**Keywords:** CAM—complementary and alternative medicine, natural compounds, toxicity, CYP3A4, interactions

## Abstract

The use of natural compounds and, in general, the use of Complementary and Alternative Medicine (CAM), is growing steadily worldwide, both due to commercial pressure and the increasing use of self-medication and the desire to manage one’s own personal health and well-being. Patients facing a cancer diagnosis are also strongly pressured to use these compounds, which are often added to standard therapeutic regimens, that should instead be based solely on diagnostic and therapeutic care pathways (DTCP) or evidence-based medicine (EBM). This study presents two clinical cases of cancer patients who presented to the pharmaceutical consultation service (PCD—Pharmacy Clinical Desk) established at the CRO Institute in Aviano, Italy. Both patients were using natural products along with prescribed chemotherapy. In the first case, a 55-year-old woman diagnosed with bilateral breast cancer with bone metastases, who was using natural compounds based on diosmin, escin (or aescin) and resveratrol in combination with ribociclib anticancer therapy, a severe ADR (neutropenia) was identified as a consequence of the drug–natural product interaction. In the second case, following a detailed medication review by the PCD, we avoided taking a therapeutic treatment (with natural compounds) that in itself could potentially render chemotherapy ineffective in a 57-year-old woman with multiple infiltrating ductal carcinoma of the left breast; the patient was planning to take a natural product containing St. John’s Wort tincture and lemon balm tincture, in combination with paclitaxel and trastuzumab. In addition, we describe the corrective actions taken, thus outlining the main objectives of the activity of the PCD’s pharmacy counseling service: first, to identify, report, and manage adverse drug reactions (ADRs), and second, to identify therapeutic combinations that present potential risks of toxicity or ineffectiveness of the drug therapy itself.

## 1. Introduction

The attitude toward the use of natural or herbal compounds and, in general, the use of CAM (Complementary and Alternative Medicine), has shown steady growth in recent decades in the general population. The reasons motivating the use of herbal or natural compounds are aimed at pursuing a state of greater well-being, improving personal mood and happiness, and reducing the side effects of drug therapies prescribed for the individual’s ongoing conditions at a given time [1].

The world of the Internet and, in general, the boundless availability of information obtainable through computer systems and social media, certainly increase personal awareness of illness and treatment, but they encourage self-decisions and push individuals toward self-medication. These attitudes can generate inappropriate medical treatments (at best) and the consequent worsening of the patient’s health status.

Issues related to the safety and appropriateness of use of these natural products have become more apparent in recent decades, as life expectancy has increased in general, making it more likely that concomitant diseases will occur in the same individual, resulting in adoption of “polypharmacy”, which can be defined as the use of multiple medications to be taken on the same day [2]. There is no uniform consensus about the number of drugs that define polypharmacy, but the most common definitions in the literature specify five to six drugs for polypharmacy and more than ten drugs in the same day for “excessive polypharmacy” [3,4].

In certain medical situations, such as emergency medicine, cardiac surgery, and oncology, polypharmacy is unavoidable [5]; for example, it is reported that the prevalence of polypharmacy in adults with cancer is 90% [6].

Polypharmacy itself implies patient exposure to potential inappropriate medical prescription (IMP) and risks of potential drug interactions (PDI) [7,8], which may occur at the pharmacokinetic or pharmacodynamic level, as well as increased risks of adverse drug reactions (ADRs); the potential risk increases significantly if natural compounds/CAM products taken by patient are added to conventional drug therapy [9,10,11].

Hardly ever does the individual physician have the time and tools necessary to make detailed assessment of each patient’s medication list to identify incompatibilities and IMP; in addition, all health care systems in different countries adopt different ways of delivering prescriptions and treatment services.

At the same time, however, patient care and counseling services have evolved, to provide patients with services that, on the one hand, increase the patient’s self-awareness about personal illness and ongoing therapy, and, on the other hand, help identify and manage treatment-related adverse effects. In parallel, the therapeutic approach is becoming increasingly “comprehensive” toward the patient, seeking to cover all aspects of patient care and to better understand values, awareness, compliance, and ultimately, the real therapeutic efficacy and benefit subjectively perceived by each patient.

In this context, of great importance is the activity defined as “Medication Review” (MedRev), performed by hospital pharmacists. Medication Review is described by the Pharmaceutical Care Network Europe as “a structured evaluation of a patient’s medicines with the aim of optimizing medicines use and improving health outcomes. This entails detecting drug related problems and recommending interventions” [12,13].

This activity consists of a detailed analysis of a patient’s prescription history and a current list of medications taken, assessing potential interactions, incompatibilities, risks of duplication, potential ADRs, and IMP; hospital pharmacists also assess the appropriateness of dosages based on patients’ clinical characteristics (e.g., allergies) [14]. MedRev takes on additional importance in the transitions of the same patient between different care settings or between different hospital departments [15]. The activity is not regulated uniformly among countries: for example, in the United Kingdom and the U.S.A., it is structured as a service within pharmaceutical care or by public pharmacies [16,17]; in some European countries, including Italy, it is freely organized at the regional level. MedRev can be a simple interview with the patient and the reconciliation of his or her personal drug therapy, or it can be structured with dedicated tools and algorithms [18]. A systematic review presenting various tools and methods in oncology is reported by Whitman et al. [19]. Interestingly, it can be implemented as part of research projects in Proactive Pharmacovigilance, or it can be a service of normal clinical practice exercised by structured health care personnel [20,21].

At the Centro di Riferimento Oncologico of Aviano, an oncology research and care institute in Italy, since 2015, we have created a counseling service at the Hospital Pharmacy, called PCD, the Pharmacy Clinical Desk. Cancer patients can turn to this service and ask for a comprehensive medication review, advice on their drug therapy, and assessments on the compatibility between their medication and the natural products they take or plan to take.

It is precisely through listening to and counseling patients that it has been possible to highlight the widespread use of natural products, dietary supplements, and other products.

Several reasons drive cancer patients to use these types of products, which can be defined as CAM (Complementary and Alternative Medicine) products or NPs (Natural Products), including dietary components, herbal products, homeopathic products (not registered as drugs), nutraceuticals, and similar. First, the dramatic existential situation in which patients diagnosed with cancer find themselves; second, the dissemination of information through the mass media and related commercial pressures; in addition, the exchange of communication through social media, often generated by non-experts in the field, plays a significant role. Finally, there is also a real-world context of health professionals and prescribers forcing the use of these products and not relying on evidence-based efficacy data.

The risks associated with the use of these compounds concern the potential interactions that occur, especially when they are used concurrently with chemotherapy, thus further exacerbating the inherent risks of polypharmacy. The constituents (“active ingredients”) of natural products are still chemical substances introduced into the body, thus subject to metabolism, absorption, distribution and elimination. Very often, metabolic interaction (e.g., at the CYP3A4 level) within common pathways modifies and alters therapeutic efficacy or causes toxicity to the patient. In addition, an inherent characteristic of anticancer drugs is that they have a narrow therapeutic index, and consequently, small variations in absorption or metabolism can cause harmful effects for the patient. Gathering reliable information on the clinical utility of many NPs or CAM is very difficult, scattershot, and uneven; in most cases, there is no opportunity to perform a structured search similar to that which we set up for regular drugs. Innovative tools, such as the HDI Highlighter opensource software, freely available on the Internet [22], can help expedite the identification of the text portions of published scientific research, in which there are references to herbal components, metabolic pathways, and interactions that may be clinically relevant [23]. Gougis et al., in their comprehensive review, provide us with a schematic summary of the possible interactions (known to date) between herbs, drugs, and dietary supplements involving the inhibition or induction of different cytochromes, along with a classification of the levels of evidence that support this knowledge. For example, on a scale consisting of 5 levels, level 1 is supported by multiple clinical studies reporting the interaction, whereas if there are only a few in vitro studies, the level of evidence assigned is 5 [24]. It should also be considered that the effects of an isolated plant component may differ from those of a combination product or a mixture in terms of the bioavailability of active ingredients and the resulting pharmacological effects, and this has great relevance to the ultimate effectiveness of therapy, especially to the cancer patient [25].

Herein we propose a study based on two case reports, evaluated at the PCD of the CRO Aviano, Italy, including the analysis of molecular interactions and observed clinical consequences. When adverse events are identified in individuals taking both drugs and natural products, they are almost automatically attributed—by health care professionals—to the conventional drugs themselves and rarely to the natural products; this happens because of the habit and tradition of considering the natural compounds to be totally safe or at least harmless. As a result, there is a marked under-reporting of adverse events caused by natural products in the Phytovigilance system database [26]. The two clinical cases described in this case report are an example of how a structured MedRev allows for the detection of adverse reactions due to drug-natural compounds interactions or early identification of situations that could lead to the discontinuation or ineffectiveness of cancer therapy.

## 2. Detailed Case Description: Case Reports, Dietary Supplements, and Drug–Natural Compound Interactions

### 2.1. Case Report 1

This clinical case concerns a 55-year-old woman with bilateral breast cancer with bone metastasis, with an ER/PgR positive and HER2 negative biological profile. Since May 2019, the patient has been on first-line drug treatment with ribociclib and letrozole. The treatment schedule involves taking 600 mg/day of ribociclib from day 1 to 21, followed by a 7-day break, according to a 28-day cycle, and 2.5 mg/day of letrozole continuously. On 12 October 2020, moderate neutropenia (0.85 × 10^3^/μL) occurred, resulting in a reduction in the dose of the suspect drug (ribociclib) from 600 mg/day to 400 mg/day, according to the same pattern of therapy. After dose reduction, the patient exhibited mild hematologic toxicity in subsequent cycles of therapy. The lowest neutrophil value found after dose reduction was 1.23 × 10^3^/μL. Beginning 1 July 2021, the patient started taking a dietary supplement of diosmin, escin (or aescin), and resveratrol. Two months after starting the supplement, on 8 September 2021, the patient again experienced moderate neutropenia (0.83 × 10^3^/μL). Due to serious ADRs, the supplement was discontinued permanently, and ribociclib was temporarily suspended. After the resolution of the hematologic toxicity, ribociclib was resumed at the same dosage taken previously, that is, 400 mg/day, according to the same pattern of therapy. Following the resumption of ribociclib medication and the discontinuation of the supplement, the patient again experienced neutropenia, but in a mild form. The lowest value found for neutrophils after the resumption of the drug and discontinuation of the supplement was 1.00 × 10^3^/μL. All other values found were higher than the one above.

#### 2.1.1. Dietary Supplement and Natural Compounds

The dietary supplement was taken as a self-prescription by the patient to reduce peripheral edema due to the antineoplastic drug (ribociclib) and contained diosmin, escin, and resveratrol. Specifically, each tablet contained 300 mg diosmin, 200 mg escin, and 30 mg resveratrol. The product data sheet recommends taking one tablet daily. The interview with the patient revealed that the supplement was being taken according to an indication different from that stated in the data sheet. Specifically, the patient was taking two tablets daily.

Diosmin is a natural compound that belongs to the class of flavonoids. In nature, we find large concentrations of this flavonoid mainly in citrus fruits, particularly oranges, grapefruits, and tangerines, as well as in some shrubs, such as *Ruta graveolens* and *Barosma betulina* [27]. Flavonoids are anti-inflammatory plant compounds that protect the body from free radicals.

Diosmin is mainly used in the treatment of hemorrhoids and leg sores caused by poor blood circulation [28]. It is also claimed to be able to heal several diseases, although there is no hard evidence to support these claims [29]. Diosmin is an excellent natural component that can be used alone or in combination with other flavonoids or phytochemicals.

**Figure 1:**
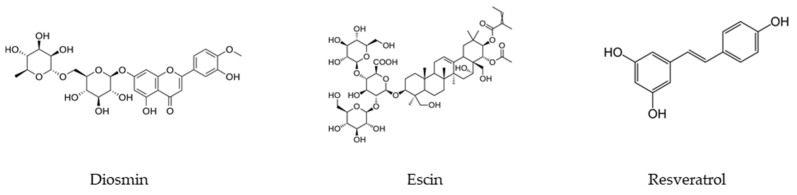
Diosmin, escin, and resveratrol structures.

Escin is a mixture of saponins contained in the seeds, bark, and leaves of horse chestnut [30]. This natural compound is known for its anti-inflammatory and anti-edematous properties, that make it useful in the treatment of chronic venous insufficiency. It also exhibits protective action on blood vessels and venotonic activity [31]. Resveratrol is a natural compound belonging to the group of stilbenoid polyphenols [32]. It is produced naturally by different plants, such as vines, blackberries, peanuts, and cocoa, for protective purposes against pathogens such as bacteria or fungi [33]. Resveratrol exerts anti-inflammatory and antioxidant effects on the body, but its main biological potential is cardioprotection. Figure 1, below, shows the structure of diosmin, escin and resveratrol.

#### 2.1.2. Antineoplastic Agents and CYP3A4 Metabolism

Ribociclib is mainly metabolized using CYP3A4 [34]. Therefore, medications that can influence CYP3A4 enzyme activity may alter the pharmacokinetics of ribociclib [35]. Concomitant use of potent CYP3A4 inhibitors should be avoided. The use of concomitant alternative drugs with lower CYP3A4 inhibitory capacity should be considered, and patients should be monitored for ribociclib-related adverse reactions (AEs). No dose modification of ribociclib is necessary at the beginning of treatment with mild or moderate CYP3A4 inhibitors [36].

In contrast, letrozole is a minor substrate of the enzyme CYP3A4. Co-administration with cimetidine, a nonspecific weak inhibitor of CYP450 enzymes, did not affect plasma concentrations of letrozole. However, the effect of potent CYP450 inhibitors is not known [37].

#### 2.1.3. Drug–Natural Compound Interactions: CYP3A4 Inhibition

The currently available data on CYP3A4 inhibition by diosmin are not completely consistent. Although the summary of product characteristics for the diosmin formulation indicates safe administration and no drug–drug interactions, there are several studies indicating an interaction of diosmin with several cytochrome P450 (CYP) isoenzymes and the drug transporter P-glycoprotein (P-gp) [38,39]. These results indicate that flavonoids, particularly diosmin, have the potential to inhibit the CYP3A4 enzyme and interact with other drugs and medications. A 2016 study showed that taking 500 mg/day of diosmin during carbamazepine therapy was found to inhibit drug metabolism through inhibition of the CYP3A4 enzyme [40]. Carbamazepine as well as the drug ribociclib represent major substrates of CYP3A4. However, to date, the data are incomplete and rather conflicting, complicating the assessment of the risk of natural drug–compound interactions when consuming diosmin [41].

Horse chestnut seed extract (HCSE) derived from *Aesculus hippocastanum* was found to inhibit CYP3A4 activity [42]. A rat study evaluated the effect of escin on the CYP450 enzyme in vivo. The results suggest that escin has an inhibitory action on CYP3A4, causing a natural drug–compound interaction with the CYP3A4 substrate [43].

Resveratrol is a natural compound with potent CYP3A4 inhibitory action. The ability of resveratrol to inhibit CYP3A4 both in vitro and in vivo has been well demonstrated. Studies in rats have shown that oral intake of resveratrol in two different dosages (2.5 or 10 mg/kg body weight) can inhibit CYP3A4 activity. The inhibition of the enzyme resulted in an increase in the bioavailability of the drugs under study (diltiazem and nicardipine)—hence the need for the dosage adjustment of therapies when taking resveratrol [44,45].

It has also been suggested that resveratrol may act as an irreversible, mechanism-based inactivator of the CYP3A4 enzyme [46,47]. This mechanism-based inhibition occurs when a CYP3A4 substrate/inhibitor forms a reactive intermediate in the active site of CYP3A4, leading to the inactivation of the enzyme through heme or apoprotein modification [48]. Because resveratrol has low bioavailability, it appears that intestinal CYP enzymes are predominantly inhibited by resveratrol. Therefore, resveratrol could impair the pharmacokinetics of some drugs, particularly those with a high intestinal first-pass effect, leading to increased toxicity or decreased activity [49]. Therefore, administration in patients undergoing drug treatment should be studied in terms of possible interactions, especially with drugs metabolized by the CYP3A4, CYP2C9, and CYP2D6 isoenzymes [50].

#### 2.1.4. Actions Taken

The oncologist, after the onset of moderate neutropenia, requested advice from our Pharmacy Clinical Desk (PCD) about possible interactions between the patient’s therapy and the previously discussed supplement. Our evaluation revealed CYP3A4 inhibitory properties by the three natural compounds in the supplement. This inhibition, as already seen, could be responsible for an increased risk of exposure to the typical side effects of ribociclib. It should also be noted that the patient was not only taking an ill-advised supplement in conjunction with drug therapy but also double the recommended dose, thus exacerbating the risk of possible side effects. For this reason, the supplement was permanently discontinued following our advice, as well as given the hematologic toxicity developed by the patient.

After providing the assessment to the oncologist, the detected adverse reaction was correctly reported to the Italian Phytovigilance system database through the VigiErbe online portal [51]. In the report, the supplement taken by the patient was included as a suspect, reporting the composition, the dosages of individual natural components, and the mode of intake. In addition, ribociclib was included as a suspect drug because neutropenia is a very common adverse drug reaction (≥1/10) [35]. The Italian National Institute of Health, which carried out the causality evaluation of the form, defined the correlation between the adverse reaction neutropenia and the natural substances considered suspect as possible. In particular, it states that it cannot be excluded that the suspected product, taken in conjunction with cancer therapy, had a modifying effect on the pharmacokinetics of the drug ribociclib, thus contributing to the onset of neutropenia.

It can be concluded, therefore, that an assessment of possible drug–natural compound interactions carried out before taking the supplement would have led us to advise against taking the product with possible reduced exposure to side effects.

### 2.2. Case Report 2

This individual case concerns a 57-year-old woman with multiple infiltrating ductal carcinoma of the left breast, associated with foci of high-grade intraductal carcinoma and ER/PgR positive biological profile. Since May 2022, the patient has been on drug treatment with paclitaxel and trastuzumab. The treatment schedule includes a weekly infusion of 80 mg/mq of paclitaxel and trastuzumab, according to a 21-day cycle. As of 26 July 2022, the patient continued therapy with only trastuzumab q21.

#### 2.2.1. Dietary Supplement and Natural Compounds

The patient presented to the Pharmacy Clinical Desk to request an evaluation of a self-prescribed supplement to reduce chemotherapy stress. The supplement contained 31 g of mother tincture of St. John’s Wort (*Hypericum perforatum*) and 31 g of mother tincture of lemon balm (*Melissa officinalis* L.). The product data sheet recommended taking 15 drops three times a day.

**Figure 2:**
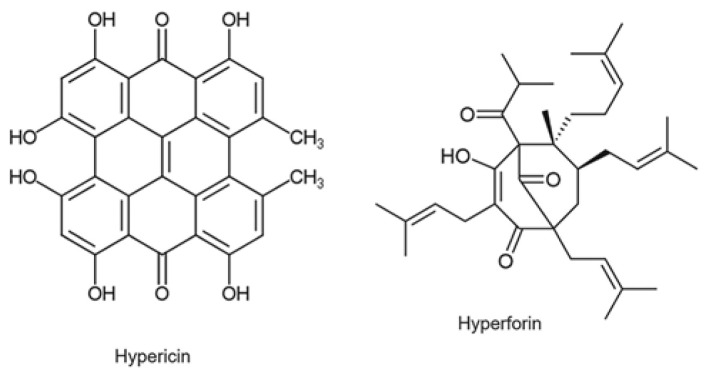
Hypericin and hyperforin structures.

St. John’s Wort is a yellow-flowered plant that has been used in traditional European medicine since the ancient Greeks [52]. St. John’s Wort contains dozens of biologically active substances, although the greatest medicinal activity is related to the presence of hypericin (a naphthodanthrone) and hyperforin (a lipophilic Phloroglucinol). Other compounds, including the flavonoids rutin, quercetin, and kaempferol, also appear to have medicinal activity [53]. In mild and moderate depression, St. John’s Wort has shown greater efficacy than placebo and equal efficacy to standard antidepressant drugs. There are no data, however, to support its use for severe depression and for periods longer than 12 weeks [54]. Studies have also been conducted to test the efficacy of St. John’s Wort concerning pathological conditions other than depression. For chronic hepatitis C virus (HCV) infection, HIV infection, and social anxiety disorder the evidence to date suggests that St. John’s Wort is not useful. It might, however, be useful in treating menopausal symptoms, wound healing, and somatic symptom disorders. However, the data to date are inconsistent to establish this with certainty [55]. Commercially, St. John’s Wort is available as a formulation for herbal teas, tablets, mother tinctures, and topical preparations. Figure 2, below, shows the structure of hypericin and hyperforin.

*Melissa officinalis* L. is a medicinal plant traditionally used in various ethno-medical systems, particularly in Traditional European Medicine and Traditional Iranian Medicine for the treatment of various diseases. It is also widely used as a vegetable and to flavor dishes [56]. Biological studies have shown that essential oil and extracts of lemon balm have natural components capable of determining pharmacological effects with potential clinical uses. The main active constituents of lemon balm include volatile compounds (geranial, neral, citronellal, and geraniol), triterpenes (ursolic acid and oleanolic acid), phenolic acids (rosmarinic acid, caffeic acid, and chlorogenic acid), and flavonoids (quercetin, rhamnocitrin, and luteolin) [57]. The use of lemon balm leaves for symptoms related to mild stress, insomnia, and mild digestive disorders is based on the traditional use of the plant. Although, however, there is insufficient evidence to support its efficacy in these clinical settings, its effectiveness seems plausible. Lemon balm has been used for these issues for at least 15 years within the EU as the intended uses do not require medical consultation [58].

#### 2.2.2. Antineoplastic Agents and CYP3A4 Metabolism

Paclitaxel is mainly metabolized by CYP2C8 and CYP3A4 [59]. The concomitant administration of paclitaxel with drugs known to induce both CYP2C8 and CYP3A4 is not recommended because efficacy may be compromised due to reduced exposure to paclitaxel [60].

#### 2.2.3. Drug–Natural Compound Interactions: CYP3A4 Inhibition

St. John’s Wort is known to induce the hepatic cytochrome P450 enzyme system. Specifically, this plant induces the expression of an isoform of the P450 enzyme system known as CYP3A4 [61]. This enzymatic cascade is harnessed by the body to break down a range of drugs and toxins. More than half of currently used chemotherapeutic agents are metabolized by the cytochrome system. Antineoplastic agents that undergo metabolism by the liver mediated by the P450 system include vinca alkaloids and the drugs etoposide, teniposide, anthracycline, paclitaxel, docetaxel, and tamoxifen [62]. In vitro and in vivo studies have demonstrated the herb–drug interaction potential of St. John’s Wort. Therefore, the concomitant intake of St. John’s Wort with several CYP substrates has been studied in humans. Some clinical cases have shown a significant herb–drug interaction between St. John’s Wort and prescription drugs [63]. Specifically, St. John’s Wort alters the pharmacokinetics of several CYP3A4 and P-gp substrates, including omeprazole, simvastatin, cyclosporine, indinavir, verapamil, and tacrolimus [64]. Furthermore, from research on the use of St. John’s Wort for depression and its interactions with drugs, it has been clearly shown that it can interact dangerously, sometimes lethally, with various substrates [65]. In contrast, no pharmacokinetic alterations from lemon balm involving CYP3A4 have emerged from the available scientific literature.

#### 2.2.4. Actions Taken

The PCD evaluation revealed a relevant clinical interaction between paclitaxel and the subject supplement. Specifically, St. John’s Wort appears to be a natural component with strong inductive activity on the enzyme CYP3A4, of which paclitaxel is a substrate. This could, therefore, result in reduced therapeutic efficacy of paclitaxel. For this reason, taking the supplement was not recommended. In contrast, no interactions with lemon balm were found. Fortunately, the patient approached the service before taking the supplement. This limited the potential risk of reduced drug activity.

## 3. Discussion

The two cases analyzed in this study report the possible lack of efficacy of antineoplastic therapy or the occurrence of ADRs (adverse drug reactions). In particular, interactions related to the self-administration of dietary supplements or natural compounds, to mitigate some of the common adverse effects of chemotherapy, have emerged [66]. In both cases, these were natural drug–compound interactions but only in the first case presented had the patient already taken the natural compound that led to the onset of the adverse effect. In the other case, however, the evaluation was carried out before taking the supplement, thus limiting the potential reduction in drug activity. Specifically, the two interactions noted and reported involved oncology drug metabolism and, specifically, CYP3A4 inhibition or induction. Briefly, the natural drug–compound interactions identified are as follows: (1) inhibitory action of diosmin, escin, and resveratrol on CYP3A4 resulting in increased plasma concentrations of the antineoplastic agent and increased risk of exposure to ADRs; (2) the enzymatic induction of St. John’s Wort on CYP3A4, resulting in the reduced pharmacological action of paclitaxel.

**Figure 3:**
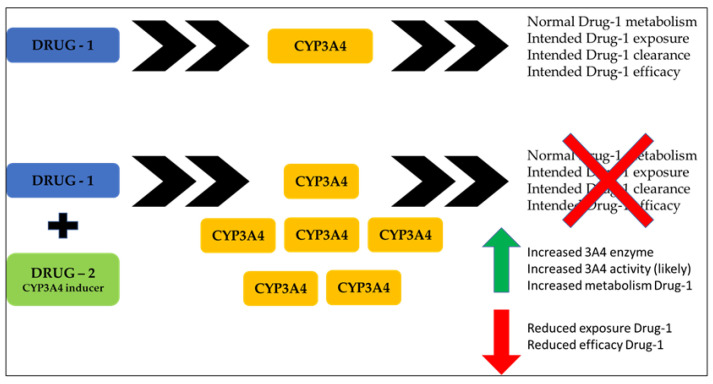
CYP3A4 activity and drug metabolism. After drug interaction, normal metabolism is impaired (red cross). An induction (green arrow) of CYP3A4 increases its activity, reducing exposure to drug 1 and its efficacy (red arrow).

Specifically, cytochromes P450 (CYP) are a superfamily of enzymes that catalyze a wide range of substrates [67]. CYP3A4 is one of the major cytochrome P450s. In fact, of all the CYP enzymes, CYP3A4 is the most important and the most abundant in both the liver and the intestinal tract [68]. It catalyzes a wide range of substrates and is responsible for the oxidation of more than 50% of commonly used drugs. Table 1, below, shows a partial list of CYP3A4 substrates [69]. CYP3A4 inhibition can be of two types: reversible and irreversible. The latter involves the inactivation of the enzyme through the formation of metabolic intermediates that irreversibly bind to and inactivate the enzyme. The clinical effects of an irreversible inhibitor are more pronounced after multiple drug administration and last longer than those of a reversible inhibitor [70]. CYP3A4 inhibition may lead to toxicities and interactions. In some cases, however, CYP3A4 inactivation may be beneficial because it may improve the therapeutic efficacy of rapidly metabolized drugs by increasing their plasma levels [71]. Unlike inhibition, induction occurs more slowly. It also takes longer for induction to stop affecting drug metabolism. For example, the induction of CYP3A4 by rifampin takes about six days to develop and eleven days to disappear [72]. Induction normally results in a decrease in drug effect. However, it can lead to increased toxicity if the increased metabolism of the main compound is accompanied by increased exposure to a toxic metabolite [73]. In Figure 3 below, Drug 1 is metabolized by CYP3A4. However, if the patient starts taking another drug that is a CYP3A4 inducer, the increase in the amount of CYP3A4 will likely lead to an increase in CYP3A4 activity and Drug 1 metabolism. This will cause a reduction in drug exposure and thus a reduction in efficacy, as shown in Figure 3 below. Table 1, below, also shows a partial list of CYP3A4 inhibitors and inducers.

## 4. Conclusions

There is a strong push for the use of alternative and complementary substances by cancer patients. Self-medication is favored by easy access to information in the age of the internet and social media. However, there is no corresponding awareness, even on the part of healthcare professionals, about the risks involved in the free and uncontrolled intake of such products. These risks not only compromise therapeutic efficacy, which is essential in cancer treatment but also expose patients to the potential risk of developing toxicity and adverse drug reactions. This risk should be associated with the tendency of physicians to underestimate the relevance of adverse reactions, and for this reason, there is a strong underreporting of individual cases in Pharmacovigilance international reporting systems [66]. In addition, an oncologist does not usually have time to listen in detail to the patient’s narrative, who sometimes trusts a pharmacist more easily. This is why we consider the PCD counseling experience at the Aviano CRO an innovative proposal.

It is an extremely impactful tool for clinical practice, as a specific control toward risks from drug combinations with natural and alternative substances (CAM) taken by patients. The two clinical cases presented were identified during a recent local monitoring study of PCD activity at the Aviano CRO (data under review and submission) that included 275 patients, with follow-up and monitoring for 24 months (2019–2022). A total of 150 adverse drug reactions were detected, registered in the Italian Pharmacovigilance reporting system (AIFA), and 762 near-misses were detected, resulting from drug–drug interactions (87%), inappropriate use of drugs (7.9%), inappropriate prescription (4.4%), and incorrect doses (0.7%); five additional adverse reactions, directly resulting from natural products, were detected and recorded in the Italian Phytovigilance system database (VigiErbe). The re-evaluation of therapy, including drug modification/suspension and monitoring over time, was applied after medical consultation. In all cases, clinicians were always informed, so that treatment choices or the actual modification of therapy were handled appropriately by the clinician himself, ensuring patient information and empowerment. Evaluating the composition of the supplement and the presence of any natural drug–compound interactions before intake may reduce the risk of reduced drug efficacy or increased risk of exposure to adverse events. In this way, the patient’s health and course of treatment can be protected, reducing the possible risks associated with taking supplements or natural compounds. Thus, the benefit is evident not only to the patient in furthering the effectiveness of treatment but also to the physician in being able to intervene and adjust the treatment while minimizing the risks of toxicity.

## Figures and Tables

**Table 1:** Partial list of inhibitors, inducers, and substrates of CYP3A4.

Types	Example of Agents
Inhibitors	*escin* (*Aesculus hippocastanum*), amiodarone, azole antifungals, *bergamottin*, bile acids, *chamomile* (*Matricaria chamomilla* L.), cimetidine, *curcumin* (*Curcuma longa* L.), cyclosporin, diltiazem. Fluoroquinolone antibiotics, *green tea* (*Camellia sinensis*), macrolide antibiotics, *peppermint oil* (*Menta x piperita* L.), *pomegranate juice* (*citrus x paradisi*), protease inhibitors, *resveratrol*
Strong inducers	carbamazepine, *common valerian* (*Valeriana officinalis*), *Hyperforin* (*Hypericum perforatum*), glucocorticoids, phenobarbital, phenytoin, rifampicin
Substrates	alfentanyl, amiodarone, antihistamines, azole antifungal, amprenavir, benzodiazepines, calcium channel blockers, carbamazepine, codeine, cyclosporine, cyclophosphamide, erythromycin, estradiol, exemestane, HMG-CoA reductase inhibitors, hydrocortisone, imatinib, lidocaine, midazolam, ribociclib, ritonavir, sirolimus, tacrolimus, tamoxifen, testosterone, tricyclic antidepressants

## Data Availability

Not applicable.

## References

[1] Shaw D., Graeme L., Pierre D., Elizabeth W., Kelvin C. (2012). Pharmacovigilance of herbal medicine. J. Ethnopharmacol..

[2] Masnoon N., Shakib S., Kalisch-Ellett L., Caughey G.E. (2017). What is polypharmacy? A systematic review of definitions. BMC Geriatr..

[3] Fillon M. (2023). Clinicians need to stay current with polypharmacy concerns. CA A Cancer J. Clin..

[4] Mohamed M.R., Mohile S.G., Juba K.M., Awad H., Wells M., Loh K.P., Flannery M., Culakova E., Tylock R.G., Ramsdale E.E. (2023). Association of polypharmacy and potential drug-drug interactions with adverse treatment outcomes in older adults with advanced cancer. Cancer.

[5] Barak R., Waissengrin B., Wolf I. (2021). Less Is More: Polypharmacy at the End of Life. Isr. Med. Assoc. J..

[6] Novak J., Goldberg A., Dharmarajan K., Amini A., Maggiore R.J., Presley C.J., Nightingale G. (2022). Polypharmacy in older adults with cancer undergoing radiotherapy: A review. J. Geriatr. Oncol..

[7] Krečak I., Pivac L., Lucijanić M., Skelin M. (2023). Polypharmacy, Potentially Inappropriate Medications, and Drug-to-Drug Interactions in Patients with Chronic Myeloproliferative Neoplasms. Biomedicines.

[8] Ramsdale E., Mohamed M., Yu V., Otto E., Juba K., Awad H., Moorthi K., Plumb S., Patil A., Vogelzang N. (2022). Polypharmacy, Potentially Inappropriate Medications, and Drug-Drug Interactions in Vulnerable Older Adults with Advanced Cancer Initiating Cancer Treatment. Oncologist.

[9] Choudhury A., Singh P.A., Bajwa N., Dash S., Bisht P. (2023). Pharmacovigilance of herbal medicines: Concerns and future prospects. J. Ethnopharmacol..

[10] Turner J.P., McKinnon R.A., Bell J.S., Koczwara B. (2016). The Management of Polypharmacy in People with Cancer and Chronic Conditions. Cancer and Chronic Conditions.

[11] Vrijkorte E., Vries J., Schaafsma R., Wymenga M., Munnink T.O. (2020). Optimising pharmacotherapy in older cancer patients with polypharmacy. Eur. J. Cancer Care.

[12] Griese-Mammen N., Hersberger K.E., Messerli M., Leikola S., Horvat N., van Mil J.W.F., Kos M. (2018). PCNE definition of medication review: Reaching agreement. Pharm. Weekbl..

[13] PCNE Position Paper on Medication Review. https://www.pcne.org/upload/files/149_Position_Paper_on_PCNE_Medication_Review_final.pdf.

[14] Zaij S., Maia K.P., Leguelinel-Blache G., Roux-Marson C., Kinowski J.M., Richard H. (2023). Intervention of pharmacist included in multidisciplinary team to reduce adverse drug event: A qualitative systematic review. BMC Health Serv. Res..

[15] Whitman A., Erdeljac P., Jones C., Pillarella N., Nightingale G. (2021). Managing Polypharmacy in Older Adults with Cancer across Different Healthcare Settings. Drug Health Patient Saf..

[16] Medication Review in the NHS, England. https://www.england.nhs.uk/primary-care/pharmacy/smr/.

[17] Rose O., Cheong V.-L., Dhaliwall S., Eislage K., Erzkamp S., Jorgenson D., Martínez F., Luetsch K. (2020). Standards in medication review: An international perspective. Can. Pharm. J. Rev. Pharm. Can..

[18] Mackler E.R., Azar M.K., Johengen E., Farris K.B., Thompson A.N. (2022). Feasibility of a comprehensive medication review to improve medication use for patients with cancer and comorbid conditions. Support. Care Cancer.

[19] Whitman A.M., DeGregory K.A., Morris A.L., Ramsdale E.E. (2016). A Comprehensive Look at Polypharmacy and Medication Screening Tools for the Older Cancer Patient. Oncologist.

[20] Kvarnström K., Niittynen I., Kallio S., Lindén-Lahti C., Airaksinen M., Schepel L. (2023). Developing an In-House Comprehensive Medication Review Training Program for Clinical Pharmacists in a Finnish Hospital Pharmacy. Int. J. Environ. Res. Public Health.

[21] Schöttker B., Chen L.-J., Caspari R., Brenner H. (2023). Protocol of the OPTIMAL study: Optimization of polypharmacy in geriatric oncology—A randomized controlled trial. BMC Cancer.

[22] HDI Highlighter Opensource Software. https://github.com/ancnudde/hdi_highlighter.

[23] Cnudde A., Watrin P., Souard F. (2022). HDI Highlighter, The First Intelligent Tool to Screen the Literature on Herb–Drug Interactions. Clin. Pharmacokinet..

[24] Gougis P., Hilmi M., Geraud A., Mir O., Funck-Brentano C. (2021). Potential cytochrome P450-mediated pharmacokinetic interactions between herbs, food, and dietary supplements and cancer treatments. Crit. Rev. Oncol..

[25] Chan W.-J.J., Adiwidjaja J., McLachlan A.J., Boddy A.V., Harnett J.E. (2023). Interactions between natural products and cancer treatments: Underlying mechanisms and clinical importance. Cancer Chemother. Pharmacol..

[26] Orzetti S., Tommasi F., Bertola A., Bortolin G., Caccin E., Cecco S., Ferrarin E., Giacomin E., Baldo P. (2022). Genetic Therapy and Molecular Targeted Therapy in Oncology: Safety, Pharmacovigilance, and Perspectives for Research and Clinical Practice. Int. J. Mol. Sci..

[27] Huwait E., Mobashir M. (2022). Potential and Therapeutic Roles of Diosmin in Human Diseases. Biomedicines.

[28] Gerges S.H., Wahdan S.A., Elsherbiny D.A., El-Demerdash E. (2021). Pharmacology of Diosmin, a Citrus Flavone Glycoside: An Updated Review. Eur. J. Drug Metab. Pharmacokinet..

[29] Enciclopedia Humanitas. https://www.humanitas-care.it/enciclopedia/integratori-alimentari/escina/.

[30] Dudek-Makuch M., Studzińska-Sroka E. (2015). Horse chestnut–efficacy and safety in chronic venous insufficiency: An overview. Rev. Bras. Farm..

[31] Salehi B., Mishra A.P., Nigam M., Sener B., Kilic M., Sharifi-Rad M., Fokou P.V.T., Martins N., Sharifi-Rad J. (2018). Resveratrol: A Double-Edged Sword in Health Benefits. Biomedicines.

[32] Enciclopedia Humanitas. https://www.humanitas.it/enciclopedia/integratori-alimentari/resveratrolo/#:~:text=Il%20resveratrolo%20%C3%A8%20una%20sostanza,patogeni%20come%20batteri%20o%20funghi.

[33] Clinical Pharmacology. School of Medicine. https://drug-interactions.medicine.iu.edu/MainTable.aspx.

[34] Lexicomp^®^ Database. https://online.lexi.com/lco/action/home.

[35] EMA—European Medicine Agency. https://www.ema.europa.eu/en/documents/product-information/kisqali-epar-product-information_it.pdf.

[36] AIFA—Agenzia Italiana del Farmaco. https://farmaci.agenziafarmaco.gov.it/aifa/servlet/PdfDownloadServlet?pdfFileName=footer_003230_040233_RCP.pdf&sys=m0b1l3.

[37] Burkina V., Zlabek V., Halsne R., Ropstad E., Zamaratskaia G. (2016). In vitro effects of the citrus flavonoids diosmin, naringenin and naringin on the hepatic drug-metabolizing CYP3A enzyme in human, pig, mouse and fish. Biochem. Pharmacol..

[38] Yoo H.H., Lee M., Chung H.J., Lee S.K., Kim D.H. (2007). Effects of diosmin, a flavonoid glycoside in citrus fruits, on P-glycoprotein-mediated drug efflux in human intestinal Caco-2 cells. J. Agric. Food Chem..

[39] Bedada S.K., Neerati P. (2018). Modulation of CYP3A enzyme activity by diosmin and its consequence on carbamazepine pharmacokinetics in rats. Naunyn-Schmiedebergs Arch. Pharmacol..

[40] Bedada S.K., Boga P.K. (2016). Influence of diosmin on the metabolism and disposition of carbamazepine in healthy subjects. Xenobiotica.

[41] Hellum B.H., Nilsen O.G. (2008). In vitro Inhibition of CYP3A4 Metabolism and P-Glycoprotein-Mediated Transport by Trade Herbal Products. Basic Clin. Pharmacol. Toxicol..

[42] Spanakis M., Vizirianakis I.S., Mironidou-Tzouveleki M., Niopas I. In vitro inhibition of CYP3A4 and CYP2D6 activity by the horse chestnut constituents’ aescin and aesculetin. Proceedings of the 8th Southeast European Congress on Xenobiotic Metabolism and Toxicity—XEMET 2010.

[43] Huang Y., Zheng S.-L., Zhu H.-Y., Xu Z.-S., Xu R.-A. (2014). Effects of aescin on cytochrome P450 enzymes in rats. J. Ethnopharmacol..

[44] Choi J.S., Choi B.C., Kang K.W. (2009). Effect of resveratrol on the pharmacokinetics of oral and intravenous nicardipine in rats: Possible role of P-glycoprotein inhibition by resveratrol. Pharm. Int. J. Pharm. Sci..

[45] Hong S.-P., Choi D.-H., Choi J.-S. (2008). Effects of Resveratrol on the Pharmacokinetics of Diltiazem and Its Major Metabolite, Desacetyldiltiazem, in Rats. Cardiovasc. Ther..

[46] Piver B., Berthou F., Dreano Y., Lucas D. (2001). Inhibition of CYP3A, CYP1A and CYP2E1 activities by resveratrol and other non volatile red wine components. Toxicol. Lett..

[47] Zhou Z.W., Zhou S.F. (2009). Application of mechanism-based CYP inhibition for predicting drug-drug interactions. Expert Opin. Drug Metab. Toxicol..

[48] Basheer L., Schultz K., Kerem Z. (2016). Inhibition of cytochrome P450 3A by acetoxylated analogues of resveratrol in in vitro and in silico models. Sci. Rep..

[49] Detampel P., Beck M., Krähenbühl S., Huwyler J. (2012). Drug interaction potential of resveratrol. Drug Metab. Rev..

[50] Chow S., Garland L., Hsu C.H., Vining D.R., Chew W.M., Miller J.A., Perloff M., Crowell J.A., Alberts D.S. (2010). Resveratrol Modulates Drug- and Carcinogen-Metabolizing Enzymes in a Healthy Volunteer Study. Cancer Prev. Res..

[51] VigiErbe Sistema di Fitovigilanza dell’Istituto Superiore di Sanità. https://www.vigierbe.it/.

[52] Istikoglou C.I., Mavreas V., Geroulanos G. (2010). History and therapeutic properties of Hypericum Perforatum from antiquity until today. Psychiatriki.

[53] Klemow K.M., Bartlow A., Crawford J., Kocher N., Shah J., Ritsick M., Benzie I.F.F., Wachtel-Galor S. (2011). Medical Attributes of St. John’s Wort (Hypericum perforatum). Herbal Medicine: Biomolecular and Clinical Aspects.

[54] NIH—National Center for Complementary and Integrative Health. https://www.nccih.nih.gov/health/st-johns-wort.

[55] Mayo Clinic. https://www.mayoclinic.org/drugs-supplements-st-johns-wort/art-20362212.

[56] Ghazizadeh J., Sadigh-Eteghad S., Marx W., Fakhari A., Hamedeyazdan S., Torbati M., Taheri-Tarighi S., Araj-Khodaei M., Mirghafourvand M. (2021). The effects of lemon balm (*Melissa officinalis* L.) on depression and anxiety in clinical trials: A systematic review and meta-analysis. Phytother. Res..

[57] Petrisor G., Motelica L., Craciun L.N., Oprea O.C., Ficai D., Ficai A. (2022). *Melissa officinalis*: Composition, Pharmacological Effects and Derived Release Systems—A Review. Int. J. Mol. Sci..

[58] EMA/HMPC/310761/2013. Committee on Herbal Medicinal Products (HMPC). https://www.ema.europa.eu/en/documents/herbal-summary/melissa-leaf-summary-public_en.pdf.

[59] Lexicomp^®^ Database. https://online.lexi.com/lco/action/doc/retrieve/docid/multinat_f/4668446?cesid=4TfUeeaoKSW&searchUrl=%2Flco%2Faction%2Fsearch%3Fq%3Dpaclitaxel%26t%3Dname%26acs%3Dtrue%26acq%3Dpacli.

[60] AIFA—Agenzia Italiana del Farmaco. https://farmaci.agenziafarmaco.gov.it/aifa/servlet/PdfDownloadServlet?pdfFileName=footer_000829_039399_RCP.pdf&retry=0&sys=m0b1l3.

[61] Fasinu P.S., Rapp G.K. (2019). Herbal Interaction with Chemotherapeutic Drugs—A Focus on Clinically Significant Findings. Front. Oncol..

[62] Josefson D. (2002). St John’s wort interferes with chemotherapy, study shows. Br. Med. J..

[63] Wang L., Zhou G., Zhu B., Wu J., Wang J., El-Aty A.M.A., Li T., Liu J., Yang T., Wang D. (2004). St John’s wort induces both cytochrome P450 3A4–catalyzed sulfoxidation and 2C19–dependent hydroxylation of omeprazole. Clin. Pharmacol. Ther..

[64] Mai I., Störmer E., Bauer S., Krüger H., Budde K., Roots I. (2003). Impact of St John’s wort treatment on the pharmacokinetics of tacrolimus and mycophenolic acid in renal transplant patients. Nephrol. Dial. Transplant..

[65] Bilia A.R., Gallori S., Vincieri F.F. (2002). St. John’s wort and depression: Efficacy, safety and tolerability—An update. Life Sci..

[66] Baldo P., Fornasier G., Ciolfi L., Sartor I., Francescon S. (2018). Pharmacovigilance in oncology. Pharm. Weekbl..

[67] Zhang T., Zhao M., Pang Y., Zhang W., Liu L.A., Wei D.-Q. (2012). Recent progress on bioinformatics, functional genomics, and metabolomics research of cytochrome P450 and its impact on drug discovery. Curr. Top. Med. Chem..

[68] Klein K., Zanger U.M. (2013). Pharmacogenomics of Cytochrome P450 3A4: Recent Progress Toward the “Missing Heritability” Problem. Front. Genet..

[69] Li G., Yi B., Liu J., Jiang X., Pan F., Yang W., Liu H., Liu Y., Wang G. (2021). Effect of CYP3A4 Inhibitors and Inducers on Pharmacokinetics and Pharmacodynamics of Saxagliptin and Active Metabolite M2 in Humans Using Physiological-Based Pharmacokinetic Combined DPP-4 Occupancy. Front. Pharmacol..

[70] Samuels E.R., Sevrioukova I. (2018). Inhibition of Human CYP3A4 by Rationally Designed Ritonavir-Like Compounds: Impact and Interplay of the Side Group Functionalities. Mol. Pharm..

[71] Shin Y., Choi C., Oh E.S., Kim C.O., Park K., Park M.S. (2022). Effect of Rifampicin on the Pharmacokinetics of Evogliptin in Healthy Volunteers. Drug Des. Dev. Ther..

[72] Hukkanen J. (2014). Induction of cytochrome P450 enzymes: A view on human in vivo findings. Expert Rev. Clin. Pharmacol..

[73] Enciclopedia Humanitas. https://www.humanitascatania.it/enciclopedia-medica/integratori-alimentari/diosmina/.

